# Factors Influencing Customer Decisions to Use Online Food Delivery Service during the COVID-19 Pandemic

**DOI:** 10.3390/foods11010064

**Published:** 2021-12-28

**Authors:** Kyungyul Jun, Borham Yoon, Seungsuk Lee, Dong-Soo Lee

**Affiliations:** 1 Department of Food and Nutrition, Kosin University, Busan 49104, Korea; kjun@kosin.ac.kr; 2 Department of Food and Nutrition, Sunchon National University, Suncheon 57922, Korea; 3 Department of Parks, Recreation and Hospitality Administration, Arkansas Tech University, Russellville, AR 72801, USA; slee17@atu.edu (S.L.); dlee13@atu.edu (D.-S.L.)

**Keywords:** online food delivery service, COVID-19 pandemic, technology acceptance, trust, enjoyment, social influence

## Abstract

Despite the popularity of online food delivery systems in the foodservice industry, there have been few studies into customers’ decision-making process to use online food delivery services during the Coronavirus disease (COVID-19) pandemic. This study applied the technology acceptance model (TAM) to examine the factors affecting customers’ intention to use online food delivery services. Results showed (a) the perceived usefulness affects customer’s online food delivery usage directly and indirectly through customer attitude; (b) enjoyment and trust are also key factors determining behavior intention toward customer attitude using online food delivery services; (c) positive relationship between social influence and customer attitude; and (d) a positive relationship between customer attitude and behavior intention in the online food delivery service context. These findings provide theoretical and managerial implications that contribute to the online food delivery service industry.

## 1. Introduction

According to the World Health Organization (WHO), the 2019 coronavirus disease (COVID-19) erupted in China in December 2019 and expanded as a global pandemic on 11 March 2020 [1]. Because COVID-19 has a high risk of death and human-to-human transmission, self-quarantine, wearing a mask in public, social distancing, and restriction of people’s movement have been strongly recommended by WHO [1]. Consequently, most of the United States required residents to stay at home and forced foodservice operations to be closed or restricted [2]. 

With the restriction of dine-in service due to COVID-19, many restaurants adapted and heavily relied on contactless and online food delivery systems to survive. The number of foodservice and users using online food delivery systems has surged during COVID-19 [3]. About 67 percent of residents preferred using online delivery services to purchase food during the COVID-19 pandemic in the US [3]. Online food delivery service refers to internet-based food ordering delivery services that connect customers with partner foodservice operations via their websites or mobile applications [4]. Online food delivery services provide a wide range of restaurant lists, allowing customers to compare menus, prices, and even reviews from other users by restaurant types. Furthermore, the distribution of mobile devices has provided customers with a new platform—food delivery apps—that is available when they order food online. Moreover, it is expected that more customers and restaurants utilized online food delivery services during and after the COVID-19 pandemic. 

Existing studies provide an understanding of customers’ motivations to use online food delivery systems [5,6] and factors affecting online food delivery service usage [4,7,8]. Additionally, previous studies have examined the factors based on the technology acceptance model (TAM) [9] that determine whether or not an individual adapts to innovation. Although TAM is a robust and powerful theoretical framework of users’ acceptance and usage of technology, testing and extending TAM by integrating it with other factors (social influence, trust, and enjoyment) may provide insight for food service industry management to develop the strategies of online food delivery services. Moreover, few studies have examined the factors influencing customers’ decision making toward the use of online food delivery services, especially under pandemic conditions [10]. As the COVID-19 pandemic has changed customers’ dining and consumption behaviors, it is necessary to consider the COVID-19 pandemic as a context factor affecting customers’ online food delivery service [11]. Therefore, the purpose of the study is to examine the factors affecting customers’ online food delivery services usage by applying TAM and other factors (e.g., enjoyment, trust, and social influence) to provide a comprehensive model during the COVID-19 pandemic. This research provides a theoretical foundation by using the TAM in the food delivery context and practical implications for online food delivery and the foodservice industry during the COVID-19 pandemic. 

## 2. Literature Review

### 2.1. Online Food Delivery Service

The progress in informational technology has introduced a new business model into the food service industry. Along with the advent of internet technology, some big fast-food chains, especially pizza franchises, have been the pioneers to embrace online food ordering with their websites. Restaurants have adopted online food ordering because it has met or exceeded expectations in several ways for restaurant operations [12]. Online food ordering has grown in popularity among customers and restaurants because of its benefits [13].

Online food ordering through websites was introduced with several different concepts. Aside from the websites operated by restaurant chains, as mentioned above, the predecessors of online food ordering services only aggregated and listed restaurants’ names with their basic information, such as phone numbers or addresses, on their website platforms. Those platforms have begun to provide more information, including menus or prices. Subsequently, online food ordering websites have taken food orders from allied restaurants. In this stage, the food ordering platforms have grabbed the food orders solely. Restaurants took care of the delivery by themselves if delivery was available. The latest approach in the food ordering systems has been for the platform to take care of the delivery. Conclusively, when restaurants utilize online food ordering, they may operate their websites or receive the orders through multiple-restaurant platforms. In addition, the food delivery may be carried out directly by the restaurants to the customer (e.g., Domino’s), or the platform picks up the meals at the restaurant and delivers them to customers (e.g., Uber Eats). Some platforms (e.g., GrubHub) provide both services [13]. The online food delivery services began with online food ordering; the online food delivery service is separately a significant business model. Recently, online food delivery was defined as the process that food ordered online is prepared and delivered to the customers by connecting customers with partner foodservice operations via their websites or mobile applications [4].

The demand for online food delivery services has dramatically increased over the last few years and is expected to grow. The global online food delivery platform market already amounts to US $31 billion [13]. As COVID-19 has changed, customers prefer a contactless and online-to-delivery system to face-to-face and dine-in service [11,14]. The online food delivery market continues to attract new customers. Therefore, factors motivating customers to use online food delivery services under the COVID-19 pandemic are needed to understand customers’ decision-making process and therefore help the foodservice business survive in this era.

### 2.2. Technology Acceptance Model

The TAM is a widely adopted conceptual framework to explain the acceptance of new technology [9,15]. The underlying foundation of TAM is a series of concepts that explains and predicts a certain human behavior with beliefs, attitudes, and behavioral intention (BI). The relationship among belief, attitude, intention, and behavior initially provides the theoretical base to the famous and robust theories in social psychology, including the theory of reasoned action (TRA) [16] and the theory of planned behavior [17]. An individual’s attitude toward conduct is considered proportional to a weighted sum of their evaluations of relevant beliefs about the predicted consequences of that behavior in these early theories [16]. However, in TAM, general beliefs (e.g., perceived ease of use (EOU) and perceived usefulness (PU)) rather than salient beliefs are considered to play an essential role in shaping attitudes toward utilizing a particular technology [9].

One of the most significant differences between TRA and TAM is the mediator role of attitude. Attitude fully mediates the effect of beliefs on behavioral intention in TRA, so the causal relations between the beliefs (EOU and PU) are not assumed. TRA posits that a person’s attitude toward a behavior is directly proportional to the sum of the beliefs about the behavior [17,18]. Therefore, an attitude captured separately is found as a formative construct and a composite of beliefs. However, after TAM without attitude was proposed [19], many empirical studies have suggested the direct impact of beliefs on behavioral intention and the relations among beliefs (e.g., PU, EOU, enjoyment, and/or trust). In a meta-analysis review with articles in the e-commerce context, Ingham et al. [20] confirms that TAM including attitude as a mediator is a better explanatory model than TAM without attitude.

**Hypothesis** **1** **(H1).** *Attitude significantly influences behavioral intention.*

### 2.3. Perceived Usefulness (PU) and Ease of Use (EOU)

Along with attitude, TAM frequently discusses the relationships between beliefs. Attitude is explained as a partial mediator of beliefs, and EOU is claimed to be a direct causal predecessor of PU [9]. The causal relations between beliefs are proposed in many empirical studies. However, there are many inconsistencies in the relationships between beliefs. Moreover, as an example, the relations between beliefs are suggested in the reverse direction with equally persuasive logical arguments by respected scholars [21,22]. Whereas Pavlou [21] argues that trust affects EOU, Gefen et al. [22] argue that EOU affects trust. Nevertheless, Ingham et al. [20] reason the introduction of causal relationships between beliefs is based on the common usage of structural equations rather than on well-established theoretical grounds. This is a notable explanation for Davis’s [15] initial justification of the causal relationship between EOU and PU, which is more circumstantial and data-dependent than theoretical.

PU is often proposed to affect the behavioral intention directly as well as indirectly through attitude. The mediator role of PU between EOU and BI is confirmed in several reviews of TAM [23,24,25]. The direct effects of beliefs on behavioral intention were introduced in the early visions of TAM.

**Hypothesis** **2a** **(H2a).** *PU significantly influences attitude*.

**Hypothesis** **2b** **(H2b).** *PU significantly influences behavioral intention*.

**Hypothesis** **3** **(H3).** *EOU significantly influences attitude*.

### 2.4. Enjoyment (EJM)

Since Davis et al. [9] introduced the concept of enjoyment into TAM, this concept is a significant factor that drives users to use a new technology [20]. Davis et al. [9] adopted enjoyment as the extrinsic motivation to test its direct effect on the behavioral intention and the indirect effect through usefulness. Davis et al. [9] stated that whatever positive or negative feelings may be brought to mind toward a specific behavior have a causal link to intention. The direct impacts of perceived risk and trust on intention were tested by many academic studies, including Pavlou [21]. Enjoyment was driven by the motivation theory [19]. The motivation to perform an activity is broadly classified into two categories: extrinsic (instrumental) motivation and intrinsic (hedonic) motivation. Whereas perceived usefulness is an example of extrinsic motivation, enjoyment is an example of intrinsic motivation. Hederson et al. [26] argue that enjoyment is the most important predictor of intention in the study that uses TAM as the reference model for the electronic supermarket. Childers et al. [27] find that enjoyment is vital in predicting customers’ attitudes toward target behaviors. In several e-commerce studies, enjoyment is a meaningful direct predictor of the intention to use e-shopping [28,29,30,31,32] or an indirect predictor through a positive attitude toward using it [33,34]. Although many studies introduce playfulness instead of enjoyment, both enjoyment and playfulness are used similarly in empirical studies [19,31,35,36,37].

**Hypothesis** **4a** **(H4a).** *Enjoyment significantly influences attitude*.

**Hypothesis** **4b** **(H4b).** *Enjoyment significantly influences behavioral intention*.

### 2.5. Trust (TR)

Safety is one of the main reasons why many customers hesitate to purchase online [38]. Trust is the customers’ beliefs about the retailers’ safety and internet technology. In previous studies, perceived risk is treated as a distinctive variable from trust, and even the casual relationship of these two variables was studied [21]. However, they are very similar in their conceptualization except in opposite directions. Customers’ perceptions of risk are often characterized as their expectations of probable losses or other unfavorable outcomes from a transaction. Both the vendors and the transaction itself are associated with negative views [39,40]. Otherwise, trust is defined as a collection of precise ideas about the vendor’s trustworthiness [21,33,38], a sense of confidence and security about online transactions [41,42], or a combination of trustworthiness in the vendor and trustworthiness in the transaction [30,43]. Therefore, with some modifications, trust and perceived risk can be treated as alternative variables. In this study, the authors use trust as a comprehensive concept, including perceived risk.

Previous research has shown that trust directly affects intended use [38,44,45,46]. Furthermore, risk perception is a direct negative predictor of intention [21,43,47,48,49,50,51]. Trust plays an essential role in developing a good attitude towards e-shopping [35,47,49,52,53,54]. Risk perception is a direct negative predictor of attitude [47,48,49,55].

**Hypothesis** **5a** **(H5a).** *Trust significantly influences attitude*.

**Hypothesis** **5b** **(H5b).** *Trust significantly influences behavioral intention*.

### 2.6. Social Influence (SI)

According to Fishbein and Ajzen [16], subjective norms reflect how the customer is affected by the perception of some significant references to one’s behavior. According to Venkatesh et al. [56], social influence is a broad notion that encompasses the concepts of subjective norm, social factors, and image. In TAM research, social influence, including subjective norms, does not effectively predict, especially in a voluntary setting [20,53,54,55,56,57]. However, in the case of e-shopping acceptance, social influence is a direct positive antecedent of intention [26,31,42,49,58,59].

According to two research studies, social influence has a favorable effect on attitude [31,60]. Barkhi and Wallace [60] conceptualize peer influence as a salient belief to shape customer attitude toward purchasing decisions in virtual stores. Kim et al. [31] argue that subjective norms significantly affect attitude toward the use and perceived usefulness and intention to reuse in the context of customer acceptance of airline B2C e-commerce websites.

**Hypothesis** **6a** **(H6a).** *Social influence significantly influences attitude*.

**Hypothesis** **6b** **(H6b).** *Social influence significantly influences behavioral intention*.

**Figure 1:**
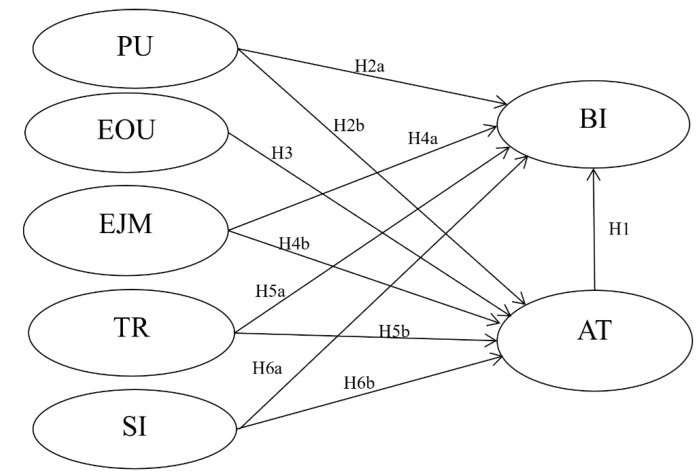
Research model. Note: PU = perceived usefulness; EOU = perceived ease of use; EJM = enjoyment; TR = trust; SI = social influence; AT= attitude; BI=behavior intention.

Based on the previous discussion, the causal relations among beliefs are set aside, and the beliefs, including PU, EOU, trust, and enjoyment, are treated as exogenous variables and modeled as attitude components toward the behavioral intention. Consequently, attitude is employed as a mediator between beliefs and intention. Also, the direct effects of beliefs on behavioral intention are allowed (Figure 1). 

## 3. Methodology

### 3.1. Measurement

A self-administered questionnaire was developed based on a comprehensive literature review of online food delivery and technology-oriented quality attributes. A total of 23 items were adopted from existing literature related to technology acceptance and online food delivery [9,17,27,29,56,61]. At the beginning of the survey, a definition of an online food delivery service was presented. The questionnaire consisted of four parts measuring the constructs including (a) TAM variables of perceived usefulness, perceived ease of use, attitude toward online food delivery service, and intention to use food delivery service; (b) trust, enjoyment, and social factors for online food delivery services; (c) experiences with online food delivery services; and (d) demographic data, including gender, ethnicity, education, and household income. Specifically, items of usefulness and ease of use were adapted from Davis [9]. Items of trust and enjoyment were utilized from Pavlou [21] and Childers et al. [27], respectively. Social influence items were taken from Ajzen [17]. Attitude items were adapted from Suh and Han [61]. Finally, behavior intention items were adapted from Suh and Han [61] and Venkatesh et al. [56]. All constructs, except for socio-demographic information, were measured using a 7-point Likert scale anchored by “strongly disagree (1)” and “strongly agree (7).” 

### 3.2. Sample and Data Collection

An online questionnaire was developed using Qualtrics and distributed via Amazon’s Mechanical Turk system (MTurk). The current study targeted the general U.S. customers over 18 years who have used online food delivery ordering during the ongoing COVID-19 pandemic. At the beginning of the survey, each participant was screened to confirm they live in the U.S. and they had at least one online food delivery ordering experience within the past three months. The data were collected through MTurk over two weeks, from 6 July 2020 to 19 July 2020. 

Among 450 responses collected, 20 respondents did not fully complete the survey. After reviewing their submissions, four samples were deleted because they failed to answer the attention check question correctly (i.e., “For this question, please select “Strongly disagree” to demonstrate your attention”). As a result of this data screening process, a total of 426 responses were used for data analysis. 

### 3.3. Data Analysis

The data were analyzed using IBM SPSS 22 and AMOS 22 software. The data analysis followed the two-step approach by Anderson and Gerbing [62]. The first step assessed the reliability and validity of the measurement model. A confirmatory factor analysis (CFA) was conducted to assess the reliability and validity of the measurement model in the first step. To evaluate reliability and validity, Cronbach’s alpha values and factor loadings were estimated. Reliability and convergence of the factors were also examined by composite reliability (CR) and average variance extracted (AVE). Discriminant validity was determined by comparing AVEs with the squared multiple correlations between constructs. 

The second step tested the research model and the proposed hypotheses by the structural equation model (SEM). Seven common model-fit measures were used to assess the model’s overall goodness of fit: the ratio of χ2 to degrees of freedom (df), comparative fit index (CFI), the goodness of fit index (GFI), adjusted goodness of fit index (AGFI), normalized fit index (NFI), Tucker-Lewis index (TLI), and root mean square error of approximation (RMSEA). 

## 4. Results

### 4.1. Descriptive Analysis

As shown in Table 1, the percentage of male respondents (54.4%) was slightly higher than the percentage of female respondents (45.5%). About 74.6% of respondents were Caucasian, followed by African American (10.1%), Asian American (7.5%), Hispanic (5.4%), Native American (0.7%), and Other (1.6%). About half of the respondents had Bachelor’s and Graduate degrees (57.0%), and nearly half of the respondents lived in suburban areas (51.9%). Approximately 77.1% of respondents used online food delivery services more than once per month.

### 4.2. Validity and Reliability of Measurements

A confirmatory factor analysis (CFA) was conducted to validate the internal and external consistency of the constructs used in the study. As shown in Table 2, the CFA results found satisfactory goodness of fit indices (χ2 = 525.962, df = 67, CMIN/df = 2.517, RMR = 0.065, GFI = 0.903, AGFI = 0.872, NFI = 0.945, IFI = 0.966, CFI = 0.966, RMSEA = 0.060) [63]. The adequacy of the measurement model was evaluated based on the criteria of reliability and convergent validity. Reliability was examined based on the composite reliability (CR) value. In Table 2, all of the values are above 0.7, indicating adequate composite reliability [63]. The average variance extracted (AVE) of all seven latent variables was higher than the suggested threshold value of 0.5, suggesting the convergent validity of the scale [63]. Thus, the reliability and convergent validity of the constructs applied in the study were supported. 

To examine discriminant validity, we compared the squared root of the AVE of each construct and its correlation coefficients with other constructs [64]. The result in Table 3 shows that all square roots of the AVEs ranging from 0.827 to 0.938 were larger than those corresponding correlation coefficients among the constructs. Thus, the discriminant validity of the constructs was supported [64]. In summary, the measurement model demonstrated adequate reliability, convergent validity, and discriminant validity.

### 4.3. Hypotheses Testing

**Figure 2:**
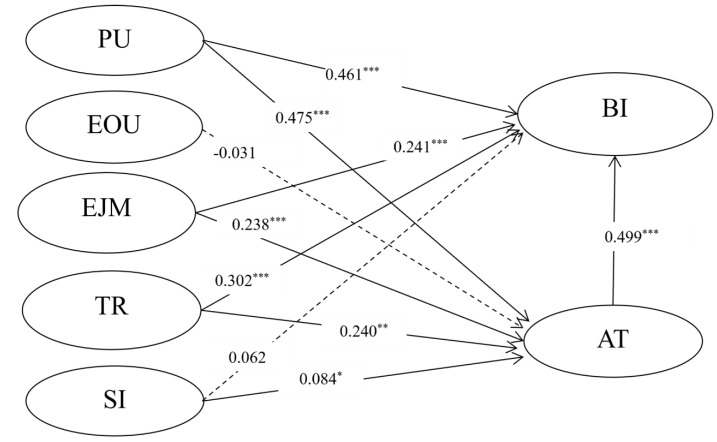
Structural equation model with parameter estimates. * *p* < 0.05, ** *p* < 0.01, *** *p* < 0.001. Non-significant paths are shown in dotted lines. Note. PU = perceived usefulness; EOU = perceived ease of use; EJM = enjoyment; TR = trust; SI = social influence; AT= attitude; BI=behavior intention.

To use the path coefficients supplied by SEM to test the hypotheses, it is necessary to assess the model’s goodness-of-fit for the variables. The goodness-of-fit tests are used to determine how well a model fits the data. The goodness-of-fit measures (χ^2^ = 526.048, df = 66, CMIN/df = 2.505, RMR = 0.065, GFI = 0.903, NFI = 0.945, IFI = 0.966, CFI = 0.966, RMSEA = 0.060) were found to largely satisfy the evaluation criteria. Figure 2 and Table 4 show the results of structural model analysis and results of testing hypotheses, respectively. Perceived usefulness (β = 0.461, *p* ≤ 0.000) was found to have a significantly positive effect on attitude (β = 0.461, *p* ≤ 0.001) and BI (β = 0.475, *p* ≤ 0.001), which means H2a and H2b were supported by the model. EOU (β = −0.031, *p* = 0.675) was not found to have a significant effect on attitude, thus not supporting H3. EJM was found to have a significant positive effect on attitude (β = 0.238, *p* ≤ 0.001) thus supporting H4a. EJM was found to have a significant effect on BI (β = 0.241, *p* ≤ 0.001), thus supporting H4b. Trust was found to positively affect attitude (β = 0.302, *p* ≤ 0.001) and BI (β = 0.240, *p* = 0.009), thus supporting H5a and H5b. Social influence was not found to affect attitude significantly, thus not supporting H6a. Social influence was found to positively affect BI (β = 0.084, *p* =0.032), thus supporting H6b. Finally, attitude was found to significantly influence BI (β = 0.499, *p* ≤ 0.001), thus supporting H1. As shown in Table 4 and Figure 2, all the hypotheses are supported except for hypotheses H3 and H6a.

## 5. Discussion

This study examined the factors affecting customers’ intentions to use online food delivery services by using the extended approach of the TAM. The findings of this study confirmed the significant effect of enjoyment (EJM), trust (TR), and social influence (SI) on customers’ acceptance of online food delivery services. Data analysis results demonstrated that perceived usefulness (PU), EJM, and TR were determinants that positively influenced BI directly. Furthermore, PU, EJM, TR, and SI were found to have an impact on BI, with attitude (AT) serving as a mediating variable. As a result, the PU, EJM, TR, SI, and AT influence the intention to use online food delivery services. According to the path analysis, it can be concluded that the proposed model in the current study fits to explain the antecedents of online food delivery service usage intention during the COVID-19 pandemic. Four of the five proposed variables (PU, EJM, TR, SI) were found as statistically significant factors influencing customers’ intention to adopt online food delivery services. 

In comparing the path coefficients of antecedents of AT, PU was the most powerful predictor of AT toward online food delivery service relative to the other belief factors. Among the factors resulting in BI, PU was found to be the most influential factor affecting customers’ online food delivery service used. This result confirms previous studies related to adopting new technologies and services in the online shopping context [20,27,34]. Moreover, these results are consistent with prior research [5,10], showing that customers are more likely to use the online food delivery service if they perceive it as useful. 

The two main beliefs in the construction of BI toward adopting new technology in the traditional form of TAM are PU and EOU. Furthermore, EOU is often used as an antecedent of PU in structural equations in recent TAM research. However, the causal relationships between beliefs are not based on well-established theoretical ground [15]. Especially in the context of e-commerce, the causal relationships among beliefs are questionable [20]. This is consistent with a finding that as attitude captured more beliefs, the more the influence of EOU declined on the model in a meta-analytic study [9].

Additionally, the current study found that EOU is not a significant factor in AT. These results are counter to previous findings [23,24,25] but consistent with results from Yuan et al. [65] and Zhao and Bacao [7]. Because customers gained enough experience from their previous usage of online food delivery services, the ease of use of online food delivery services will no longer determine customers’ attitudes after their initial adoption of online food delivery services. Furthermore, during the COVID-19 pandemic, other factors, such as safety, efficiency, and trust, are more important determinants and can provide more benefits for customers. 

The second most significant factor of customers’ online food delivery service usage intention was TR. This finding is similar to previous studies [35,38,44,45,46] showing that TR has a significant effect on customer technology adoption intention in the online shopping context. Customers may be unsure whether the restaurant accurately receives orders or whether the quality of food delivered is as excellent as the quality of food served at the restaurant, which underlines the necessity of TR in the online food delivery service context [10]. During the pandemic, when contactless delivery was essential and required, trust significantly formulated customers’ intention to use online food service under the COVID-19 pandemic situation. 

Interestingly, enjoyment was also found as a strongly significant determinant of customer attitude and intention toward using online food delivery services. This result is consistent with previous studies [35,36,37], which discovered a positive relationship between enjoyment/playfulness and attitude toward new technology in the online shopping context. This study revealed that the more customers thought that utilizing online food delivery services was exciting, fun, and enjoyable, the more they positively used those services. In the hospitality industry, hedonic motivation was the critical factor affecting customers’ service quality evaluation [66]. Likewise, customers seek excitement, pleasure, and fun when purchasing food via an online delivery service. Therefore, the current study clarified that enjoyment mattered when using the online food delivery service. Furthermore, it is a notable suggestion that these studies could replace EOU as a determinant of AT or BI in a TAM. Eventually, different beliefs influence attitude and intention to use a platform differently because different environments for the platform differentiate the importance of each belief [27].

However, the influence of SI was a statistically significant factor influencing AT, but it does not have a strong impact on BI towards online food delivery usage. These results differ from the previous findings [31] in the fashion context but are similar to previous studies [20,56,57]. Compared to the fashion products that customers tend to interact with others to reduce their uncertainty or anxiety about adopting innovation in fashion, customers’ perceived pressure is relatively low from the social community in terms of adopting or using online food delivery services.

## 6. Implications and Future Research

### 6.1. Theoretical Implications

This study contributes to the current literature with several theoretical implications. First, the present study demonstrated and provided empirical evidence that the TAM model worked in online food delivery services during the COVID-19 pandemic. The study enriched the literature on technology usage in an emergency, especially during a pandemic. With intensifying competition across the foodservice industry and increasing difficulty for companies to reach their customers during the pandemic, this study provides a theoretical framework for online food delivery literature. 

Second, this study employed the TAM to evaluate customer behavior intentions to utilize online food delivery services theoretically. The comprehensive approach of TAM with the other factors (i.e., enjoyment, trust, and social influence) is considered to have better explanatory power than the standard TAM model. This study is notable in that this study attempted to apply the TAM model to measure customer behavior, which has rarely been used in the realm of online food delivery services. Furthermore, this study confirmed that utilitarian value (i.e., perceived usefulness) is more critical than hedonic value (i.e., enjoyment) in online food delivery services used. The finding of this study makes a substantial academic contribution to e-commerce and online food services. Therefore, this study attempted to provide a comprehensive model to understand customers’ online food delivery services usage. 

### 6.2. Practical Implications

The global pandemic has limited and even obliterated in-person experiences that the food services industry depends on for survival. Many food and catering service businesses are turning to virtual events to keep their customers and generate new revenue streams [67]. Due to the restrictions on in-person service in the food service industry, customer demand for online food delivery services has skyrocketed. The COVID-19 pandemic provided numerous opportunities for customers and food service operators to adopt new technologies and develop new platforms for online food delivery. The study’s findings can help food service operators formulate effective strategies for running profitable food service businesses that use online food delivery. During the pandemic, online food delivery services improved their marketing to encourage people to use more online food services following the Centers for Disease Control and Prevention’s (CDC) social distancing guideline [1]. As a result, more jobs were created to cover the online food delivery markets. This research has managerial implications for online food delivery service providers as well. Food service operators should emphasize social influence, accurate product information, convenience, and online food service quality.

First, the study’s findings suggest that having excellent perceived usefulness is essential to promote customers’ intention to use online food delivery services. The perceived usefulness of online food delivery services could be improved by providing high quality information [8]. It is suggested that online food delivery service providers offer up-to-date information by periodically updating product information according to consumption trends (e.g., restaurant list, price, or menu information). The service providers also need to ensure that they provide accurate and reliable information, such as business hours, delivery areas, and time, which increase customers’ food ordering effectiveness.

Second, the online food delivery platform should consider providing detailed product information, which elicits enjoyment of the online service [37]. For example, service providers can offer information about the reviews of the restaurant and food, healthy menu options with calorie information, organic and locally sourced ingredients, and detailed descriptions and pictures of food, which lead customers to feel enjoyment in the food ordering process. Furthermore, the visual features and graphic design of the online food delivery service platform could provide fun for customers while using the service. 

Third, one of the most effective ways to increase customer satisfaction with online food delivery is to build customer trust. Trust is crucial in influencing positive attitudes and behaviors toward online food delivery services. Given that a firm privacy policy can increase trust in an online service [40], online food delivery service providers should commit to protecting customer privacy (e.g., personal and credit card information). Service providers should post a clearly stated privacy policy to build trust and reduce risk. 

Finally, online food delivery services managers should understand what they want and need to meet customers’ expectations during the COVID-19 pandemic. In the present pandemic, it is critical to emphasize the benefits of online food delivery services (e.g., food safety, hygiene, and contactless delivery) in reducing the annoyance caused by COVID-19 in people’s daily lives. Because of the ban on social gatherings and dine-in service, it is believed that individuals will be ready to eat at home and protect themselves during the pandemic. Therefore, food service managers must figure out how to positively reach out to their customers while persuading them to continue using online food delivery services during the pandemic. This will assist customers in perceiving highly personalized treatment from online food delivery service providers, resulting in improved business performance and increased customer satisfaction. 

### 6.3. Limitations and Future Study

Despite its implications, some limitations and suggestions for future research should be discussed. First, the sample of this study is the online food delivery service users in the United States, which may limit the generalization of the findings to other countries. Results may vary across countries due to cultural differences, technology acceptance, and other factors. As a result, the research model employed in this study should be replicated and tested in other countries to confirm its validity and usefulness. In the future, longitudinal research will be needed to fine-tune the findings of this study and compare the COVID-19 pandemic to post-COVID-19 conditions. Second, other variables such as the frequency with which an online food delivery service is used and demographic traits (such as age and gender) could be regarded as moderating factors. The relationship between variables related to TAM and customers’ intention to use online food delivery services could be different depending on the level of food delivery user experience. For example, the relationship between perceived ease of use (EOU) and attitude could be supported for customers who use online services infrequently. Due to a lack of experience, they may be unable to understand and interpret the information easily; thus, EOU could be a key factor influencing customers’ intentions to purchase food through an online food delivery platform. In future studies, it is recommended that an additional variable should be included to gain a better understanding of customer behaviors in the context of an online food delivery service.

## Figures and Tables

**Table 1:** Profile of the respondents.

Demographic Characteristics		Frequency	Percentage
Gender	Male	232	54.5
	Female	194	45.5
Ethnicity	White/Caucasian	318	74.6
	African American	43	10.1
	Hispanic or Latino	23	5.4
	Asian American	32	7.5
	Native American or American Indian	3	0.7
	Others	7	1.6
Educational level	High school diploma and under	120	28.2
	Associate degree	63	14.8
	Bachelor’s degree	180	42.4
	Graduate degree (Master or Doctoral)	62	14.6
Annual household income	Less than $20,000	47	11.0
	$20,000~$39,999	94	22.1
	$40,000~$59,999	76	17.8
	$60,000~$79,999	85	20.0
	$80,000~$99,999	50	11.7
	$100,000 or more	74	17.4
Living area	Urban	158	37.1
	Suburban	221	51.9
	Rural	47	11.0
Frequency of use	Several times a day	4	0.9
	Once a day	4	0.9
	Several times a week	68	16.0
	Once a week	105	24.6
	At least once a month	148	34.7
	At least once every two months	40	9.4
	At least once every three months	37	8.7
	Only used once	20	4.7

**Table 2:** Results of confirmatory factory analysis.

Constructs and Measurement Items	Standardized Loading	CR	AVE
Perceived Usefulness (PU, Cronbach’s Alpha = 0.871)			
Online food delivery platform makes my food ordering efficient	0.853	0.874	0.698
Online food delivery platform enhances my effectiveness in food ordering	0.814		
Online food delivery platform is useful in food ordering	0.840		
Perceived ease of use (EOU, Cronbach’s Alpha = 0.894)			
Learning to operate the online food delivery platform is easy for me	0.844	0.896	0.743
The online food delivery platform is clear and understandable	0.834		
The online food delivery platform is easy to use	0.906		
Enjoyment (EJM, Cronbach’s Alpha = 0.896)			
I have fun using the online food delivery platform	0.823	0.896	0.683
Using the online food delivery platform is exciting	0.813		
Using the online food delivery platform is enjoyable	0.854		
Using the online food delivery platform is interesting	0.816		
Trust (TR, Cronbach’s Alpha = 0.899)			
The online food delivery platform is trustworthy	0.889	0.900	0.751
The online food delivery platform keeps promises and commitments	0.833		
I trust in the online food delivery platform	0.876		
Social influence (SI, Cronbach’s Alpha = 0.902)			
People who influence my behavior think that I should use the online food delivery platform	0.870	0.904	0.759
People who are important to me think that I should use the online food delivery platform	0.944		
My friends want me to use the online food delivery platform	0.793		
Attitude (AT, Cronbach’s Alpha = 0.921)			
Using the online food delivery platform is a pleasant idea	0.889	0.921	0.795
Using the online food delivery platform is a positive idea	0.905		
Using the online food delivery platform is an appealing idea	0.881		
Behavior Intention (BI, Cronbach’s Alpha = 0.967)			
I intend to continue using the online food delivery platform in the future	0.959	0.967	0.880
I predict I would use the online food delivery platform in the future	0.932		
I plan to use the online food delivery platform in the future	0.934		
I expect my use of the online food delivery platform to continue in the future	0.927		

χ^2^/df = 2.517 (*p* < 0.001), CFI = 0.966, GFI = 0.903, AGFI = 0.872, NFI = 0.945, TLI = 0.959, RMSEA = 0.060. Note. CR = composite reliability; AVE = average variance extracted.

**Table 3:** Correlations and discriminant validity.

Variable	Perceived Usefulness	Perceived Ease of Use	Enjoyment	Trust	Social Influence	Attitude	Behavior Intention
Perceived usefulness	0.836						
Perceived ease of use	0.783	0.862					
Enjoyment	0.637	0.475	0.827				
Trust	0.799	0.724	0.675	0.866			
Social influence	0.357	0.230	0.464	0.476	0.871		
Attitude	0.809	0.642	0.728	0.801	0.466	0.892	
Behavior intention	0.763	0.615	0.499	0.725	0.406	0.763	0.938

Note: Diagonal elements show square root of the average variance extracted (AVE). Below the diagonal is the correlation coefficient.

**Table 4:** Result of structural model analysis.

Hypotheses		Beta	S.E.	Critical Ratio	*p*-Value	Decision
H1	AT -> BI	0.499 ***	0.092	5.413	0.000	Supported
H2a	PU -> AT	0.461 ***	0.086	5.366	0.000	Supported
H2b	PU -> BI	0.475 ***	0.098	4.864	0.000	Supported
H3	EOU -> AT	−0.031	0.075	−0.419	0.675	Not supported
H4a	EJM -> AT	0.238 ***	0.047	5.052	0.000	Supported
H4b	EJM -> BI	0.241 ***	0.061	−3.968	0.000	Supported
H5a	TR -> AT	0.302 ***	0.077	3.929	0.000	Supported
H5b	TR -> BI	0.240 **	0.092	2.602	0.009	Supported
H6a	SI -> AT	0.062	0.032	1.942	0.052	Not supported
H6b	SI -> BI	0.084 *	0.039	2.143	0.032	Supported

* *p* < 0.05, ** *p* < 0.01, *** *p* < 0.001. Note. PU = perceived usefulness; EOU = perceived ease of use; EJM = enjoyment; TR = trust; SI = social influence; AT = attitude; BI = behavior intention.

## Data Availability

Not applicable.
